# Supplementary material for: The role of halogens in Au–S bond cleavage for energy-differentiated catalysis at the single-bond limit
Source: Nat Commun. 2023 Nov 24;14:7695. doi: 10.1038/s41467-023-43639-8 (PMC10673828; doi:10.1038/s41467-023-43639-8)
Supplement: Supplementary file 1 — Supplementary Information [file 41467_2023_43639_MOESM1_ESM.pdf]

Supplementary Information for

The role of halogens in Au-S bond cleavage for energy-differentiated catalysis at the single-bond limit

Peihui Li[1,†], Songjun Hou[2,†], Qingqing Wu[2], Yijian Chen[1], Boyu Wang[1], Haiyang Ren[1], Jinying Wang[1], Zhaoyi Zhai[3], Zhongbo Yu*[3], Colin J. Lambert*[2], Chuancheng Jia*[1] and Xuefeng Guo*[1,4]

[1]Center of Single-Molecule Sciences, Institute of Modern Optics, Frontiers Science Center for New Organic Matter, Tianjin Key Laboratory of Micro-scale Optical Information Science and Technology, College of Electronic Information and Optical Engineering, Nankai University, 38 Tongyan Road, Jinnan District, Tianjin 300350, P. R. China.

[2]Department of Physics, Lancaster University, Lancaster, LA1 4YB, UK.

[3]State Key Laboratory of Medicinal Chemical Biology, College of Pharmacy, Nankai University, Tianjin 300350, P. R. China.

[4]Beijing National Laboratory for Molecular Sciences, National Biomedical Imaging Center, College of Chemistry and Molecular Engineering, Peking University, 292 Chengfu Road, Haidian District, Beijing 100871, P. R. China.

[†]These authors contributed equally to this work.

*Corresponding author. Email: guoxf@pku.edu.cn (X.G.); jiacc@nankai.edu.cn (C.J.); c.lambert@lancaster.ac.uk (C.J.L.); zyu@nankai.edu.cn (Z.Y.).

Table of Contents

Supplementary Note 1. Theoretical calculations for chloride catalysis

Supplementary Note 2. Theoretical calculations for iodine catalysis

Supplementary Note 3. Molecular structures of AC–SAc and OPE3–SAc

Supplementary Note 4. Detailed experimental conductance results of the AC–SAc molecule in dodecane

Supplementary Note 5. Detailed experimental conductance results of the AC–SAc molecule in TMB

Supplementary Note 6. Detailed experimental conductance results of the AC–SAc molecule in TCB

Supplementary Note 7. Separately displayed experimental results for the AC–SAc molecule in TCB

Supplementary Note 8. Experimental conductance measurements of the AC–SH molecule in TCB

Supplementary Note 9. Calculated transmission spectra of the single-molecule junction with –SAc and –SH anchors

Supplementary Note 10. Experimental conductance measurements of the OPE3–SAc molecule

Supplementary Note 11. Separately displayed experimental results for the OPE3–SAc molecule

Supplementary Note 12. The origin of the discontinuities in the energy-displacement curve

Supplementary Note 13. Single-molecule force spectroscopy measurement of 1,8-octanedithiol (C8)

Supplementary Note 14. Energy evolution during Au–S bond cleavage in the absence of halogen catalysis

Supplementary Note 15. Evolution of energy and Voronoi charge as the distance between gold tip atoms and chlorine atoms

Supplementary Note 16. Experimental results with the AC–SAc molecule in TBB/TCB at 0.1 V

Supplementary Note 17. The discussion of the semiconductive properties of halogen-metal complexes

Supplementary Note 18. Theoretical molecular models for energy evolution calculation

Supplementary Note 19. The final states of halogen atoms (Cl and I) and the topmost gold atom

Supplementary Note 1. Theoretical calculations for chloride catalysis

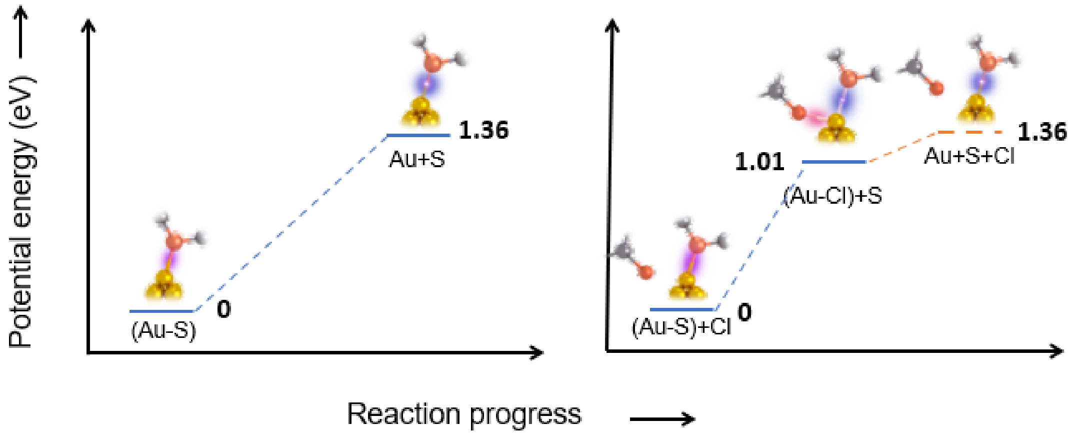

Supplementary Fig. 1 | Energy diagrams for Au–S bond breaking without (left) and with (right) chloride catalysis. In the calculation, the anchor S is constructed based on −SAc group, and the Cl comes from 1,2,4-trichlorobenzene (TCB). The structure is optimized.

Supplementary Note 2. Theoretical calculations for iodine catalysis

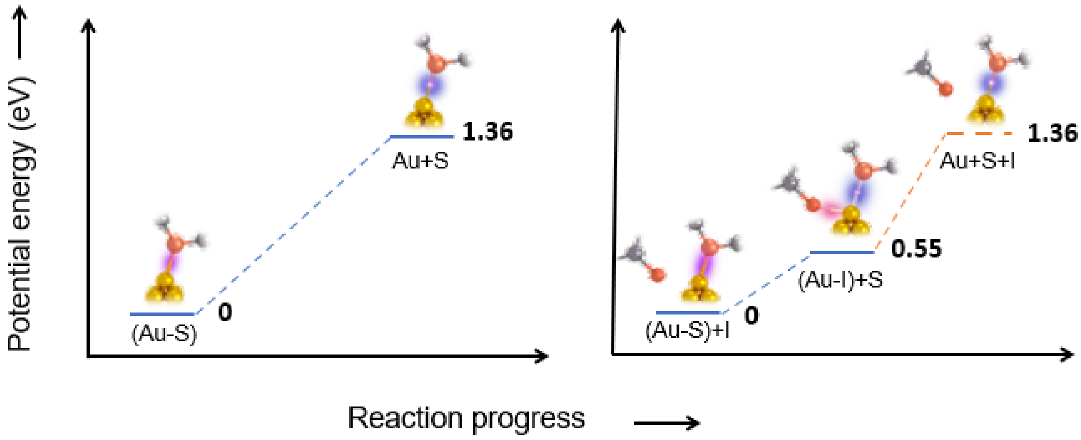

Supplementary Fig. 2 | Energy diagrams for Au–S bond breaking without (left) and with (right) iodine catalysis. In the calculation, the anchor S is constructed based on −SAc group, and the I comes from triiodobenzene (TIB). The structure is optimized.

Supplementary Note 3. Molecular structures of AC–SAc and OPE3–SAc

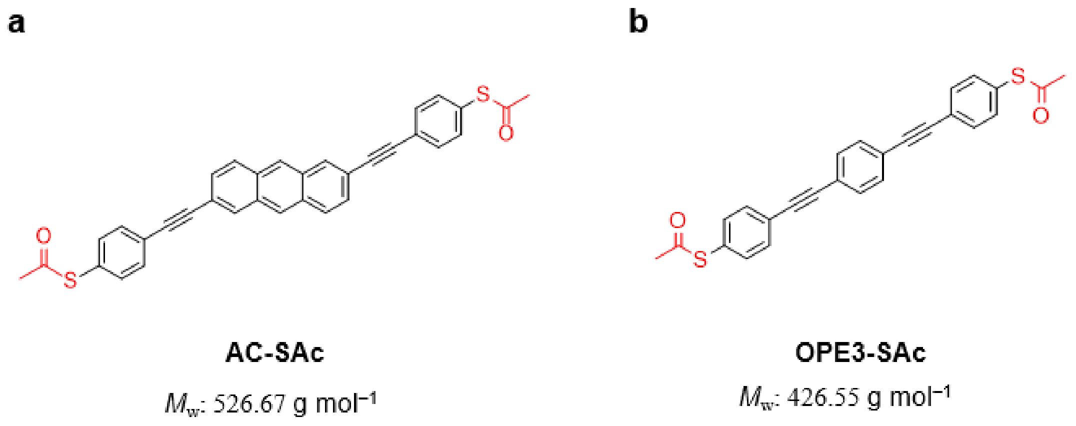

Supplementary Fig. 3 | Molecular structures. a, b Structures of AC–SAc and OPE3–SAc molecules.

Supplementary Note 4. Detailed experimental conductance results of the AC–SAc molecule in dodecane

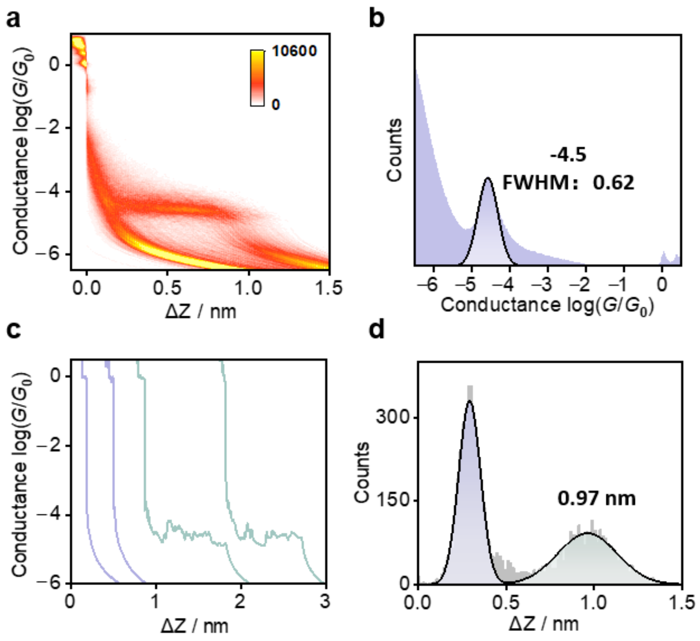

Supplementary Fig. 4 | Experimental results of the AC–SAc molecule in dodecane at 0.1 V. a, 2D conductance-displacement histograms of the AC–SAc molecule. The X axes is divided into 500 parts, that is, bin size of X is 500. While the Y axes is divided into 1000 parts, that is, bin size of X is 1000. Therefore, there are 1000*500 grids. All the single traces are superimposed in this 2D diagram. Whenever a point falls on any grid, the number in that grid will increase by one. The color bar means the number of points in corresponding grid. b, 1D conductance histograms of the AC–SAc molecule. c, Typical single conductance-displacement traces. d, Plateau length of the conductance state of the AC–SAc molecule.

Supplementary Note 5. Detailed experimental conductance results of the AC–SAc molecule in TMB

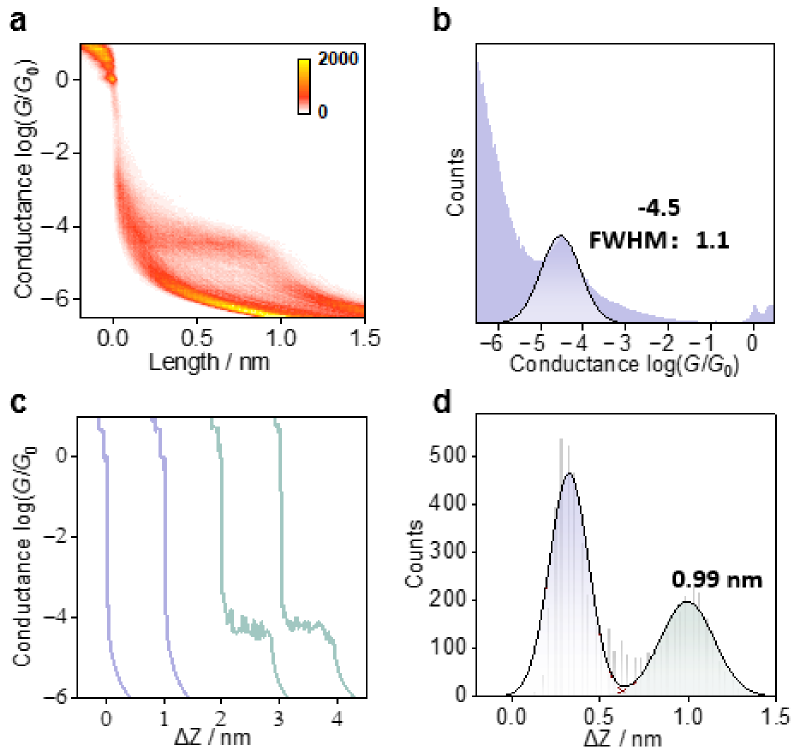

Supplementary Fig. 5 | Experimental results of the AC–SAc molecule in TMB at 0.1 V. a, 2D conductance-displacement histograms of the AC–SAc molecule. The X axes is divided into 500 parts, that is, bin size of X is 500. While the Y axes is divided into 1000 parts, that is, bin size of X is 1000. Therefore, there are 1000*500 grids. All the single traces are superimposed in this 2D diagram. Whenever a point falls on any grid, the number in that grid will increase by one. The color bar means the number of points in corresponding grid. b, 1D conductance histograms of the AC–SAc molecule. c, Typical single conductance-displacement traces. d, Plateau lengths of the conductance state of the AC–SAc molecule.

Supplementary Note 6. Detailed experimental conductance results of the AC–SAc molecule in TCB

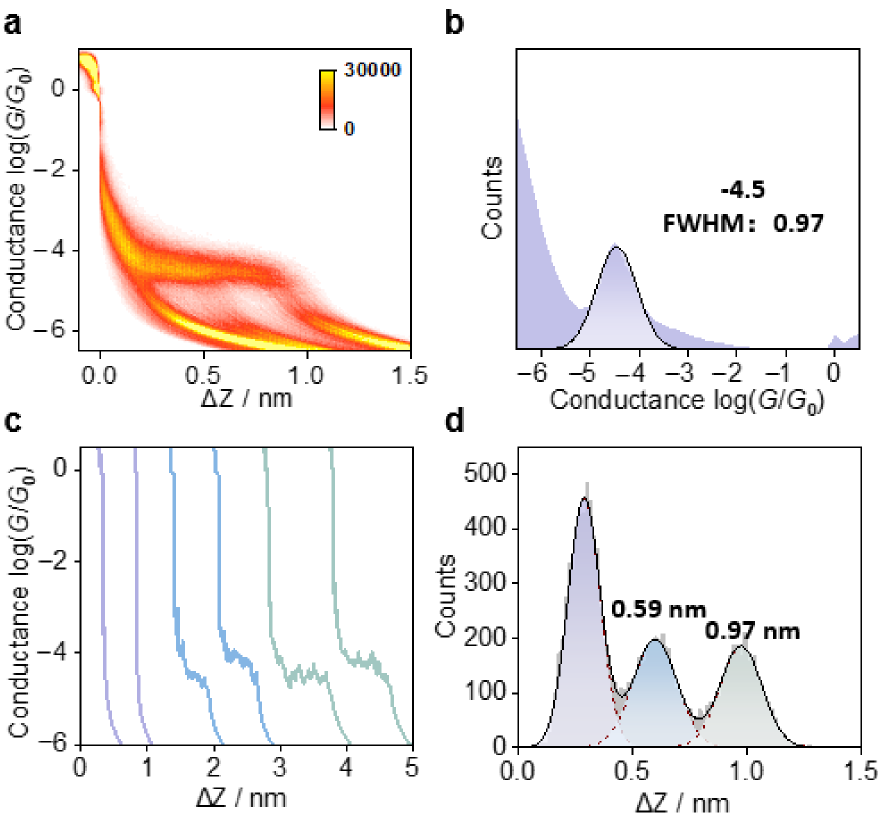

Supplementary Fig. 6 | Experimental results of the AC–SAc molecule in TCB at 0.1 V. a, 2D conductance-displacement histograms of the AC–SAc molecule. The X axes is divided into 500 parts, that is, bin size of X is 500. While the Y axes is divided into 1000 parts, that is, bin size of X is 1000. Therefore, there are 1000*500 grids. All the single traces are superimposed in this 2D diagram. Whenever a point falls on any grid, the number in that grid will increase by one. The color bar means the number of points in corresponding grid. b, 1D conductance histograms of the AC–SAc molecule. c, Typical single conductance-displacement traces. d, Plateau lengths of the conductance state of the AC–SAc molecule.

Supplementary Note 7. Separately displayed experimental results for the AC–SAc molecule in TCB

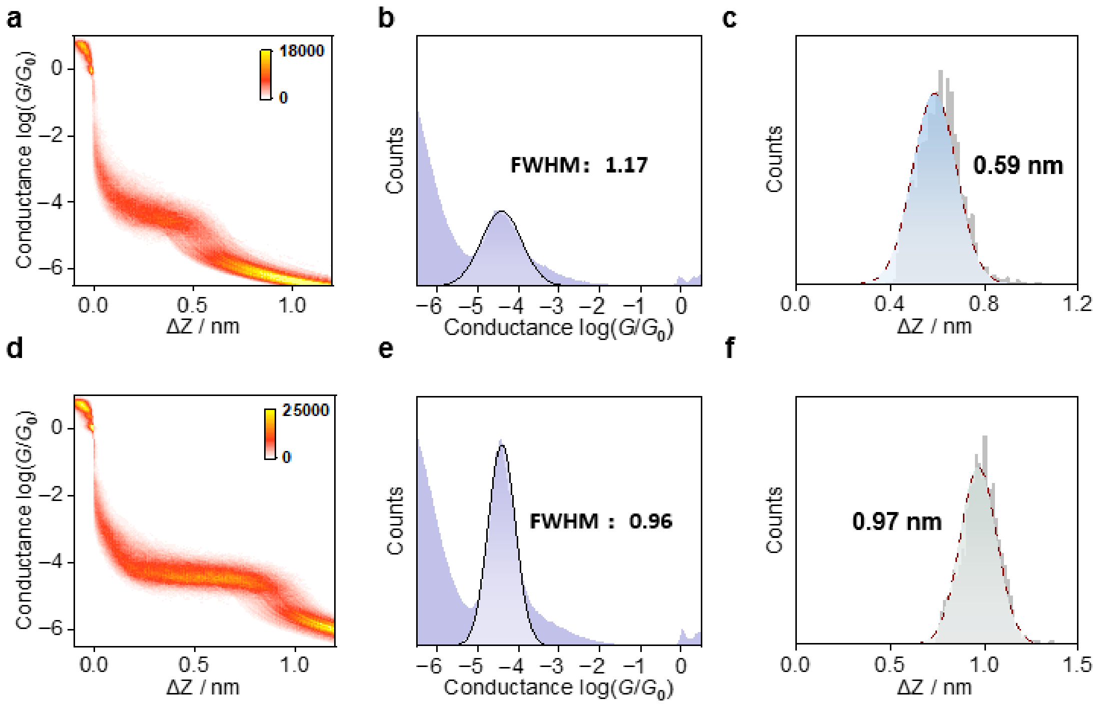

Supplementary Fig. 7 | Separated short and long plateau length states of the AC–SAc molecule at 0.1 V. 2D conductance-displacement histograms of the short plateau length state (a) and the long plateau length state (d). The X axes is divided into 500 parts, that is, bin size of X is 500. While the Y axes is divided into 1000 parts, that is, bin size of X is 1000. Therefore, there are 1000*500 grids. All the single traces are superimposed in this 2D diagram. Whenever a point falls on any grid, the number in that grid will increase by one. The color bar means the number of points in corresponding grid. 1D conductance histograms of the short plateau length state (b) and long plateau length state (e). Plateau lengths of the short plateau length state (c) and the long plateau length state (f).

Supplementary Note 8. Experimental conductance measurements of the AC−SH molecule in TCB

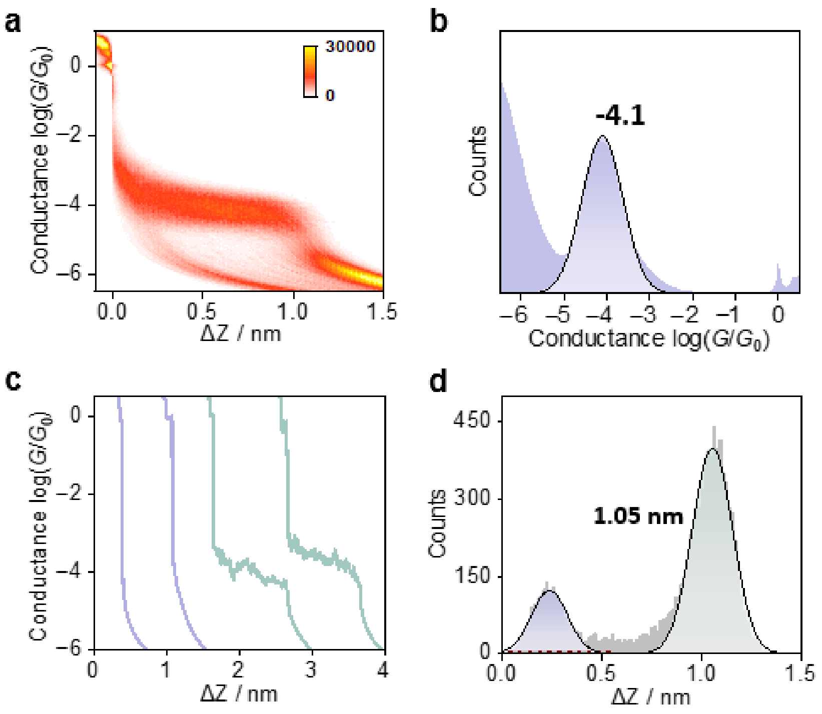

Supplementary Fig. 8 | Experimental results of the AC−SH molecule in TCB at 0.1 V. a, 2D conductance-displacement histograms of the AC−SH molecule. The X axes is divided into 500 parts, that is, bin size of X is 500. While the Y axes is divided into 1000 parts, that is, bin size of X is 1000. Therefore, there are 1000*500 grids. All the single traces are superimposed in this 2D diagram. Whenever a point falls on any grid, the number in that grid will increase by one. The color bar means the number of points in corresponding grid. b, 1D conductance histograms of the AC−SH molecule. c, Typical single conductance-displacement traces. d, Plateau lengths of the conductance state of the AC−SH molecule.

Supplementary Note 9. Calculated transmission spectra of the single-molecule junction with –SAc and –SH anchors

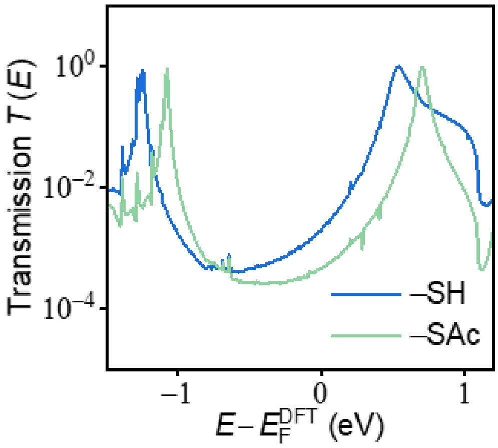

Supplementary Fig. 9 | Calculated transmission spectra of the single-molecule junction with –SAc and –SH anchors.

Supplementary Note 10. Experimental conductance measurements of the OPE3–SAc molecule

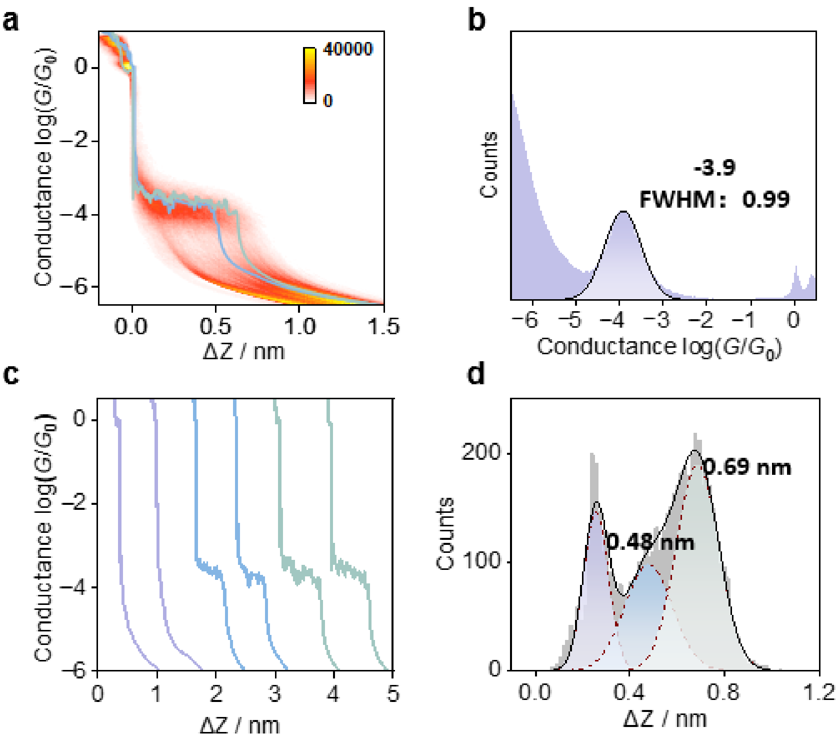

Supplementary Fig. 10 | Experimental results of the OPE3–SAc molecule in TCB at 0.1 V. a, 2D conductance-displacement histograms of the OPE3–SAc molecule. The X axes is divided into 500 parts, that is, bin size of X is 500. While the Y axes is divided into 1000 parts, that is, bin size of X is 1000. Therefore, there are 1000*500 grids. All the single traces are superimposed in this 2D diagram. Whenever a point falls on any grid, the number in that grid will increase by one. The color bar means the number of points in corresponding grid. b, 1D conductance histograms of the OPE3–SAc molecule. c, Typical single conductance-displacement traces. d, Plateau length of the conductance state of the OPE3–SAc molecule.

Supplementary Note 11. Separately displayed experimental results for the OPE3–SAc molecule

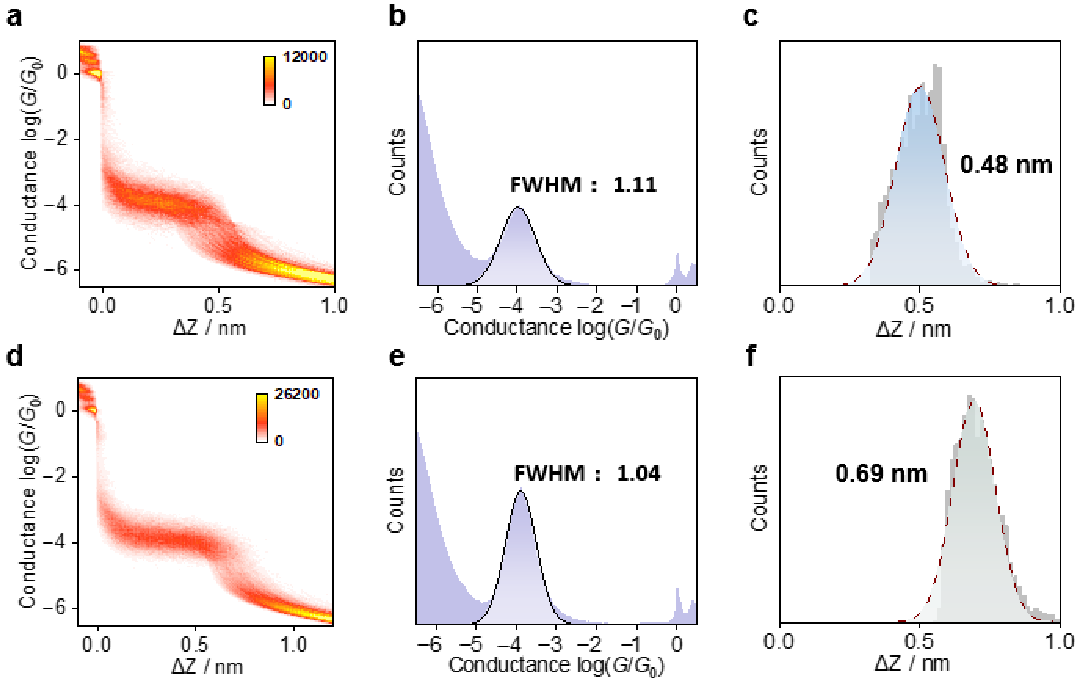

Supplementary Fig. 11 | Separated short and long plateau length states of the OPE3–SAc molecule in TCB at 0.1 V. 2D conductance-displacement histograms of the short plateau length state (a) and the long plateau length state (d). The X axes is divided into 500 parts, that is, bin size of X is 500. While the Y axes is divided into 1000 parts, that is, bin size of X is 1000. Therefore, there are 1000*500 grids. All the single traces are superimposed in this 2D diagram. Whenever a point falls on any grid, the number in that grid will increase by one. The color bar means the number of points in corresponding grid. 1D conductance histograms of the short plateau length state (b) and long plateau length state (e). plateau lengths of the short plateau length state (c) and the long plateau length state (f).

Supplementary Note 12. The origin of the discontinuities in the energy-displacement curve

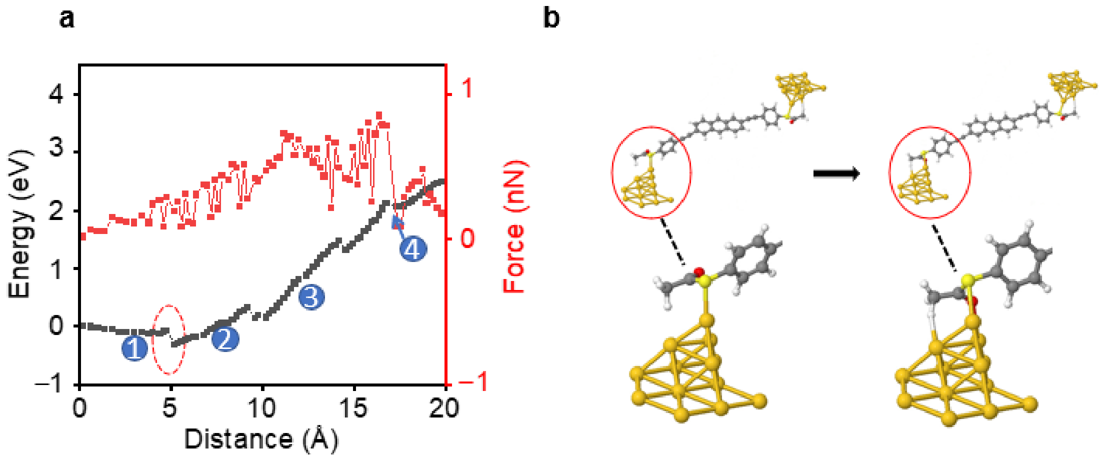

Supplementary Fig. 12 | The origin of the discontinuities in the energy-displacement curve. a, The energy-displacement curve. The first discontinuity is indicated by the red dash circle. b, The geometries of two adjacent steps corresponding to the discontinuity shown in (a). The parts with a significant change are zoomed in, which suggests that the H and O atoms in Ac group are attached to gold atoms, resulting in a lower energy.

Supplementary Note 13. Single-molecule force spectroscopy measurement of 1,8-octanedithiol (C8)

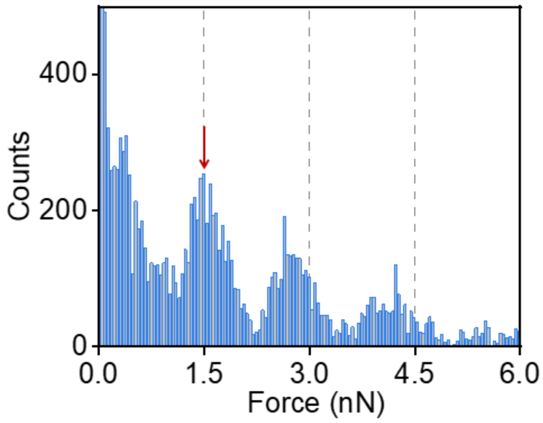

Supplementary Fig. 13 | Force histograms for C8. The results for a standard molecule C8, which is used as internal standard for force measurements. Considering the systematic error from the AFM tip, instrument, cell and so on, the single-molecule force of C8 was measured and calibrated according to the results in the literature[1]. Then, the calibration parameter was applied to the measurement of OPE3–SAc in TCB solution and in dodecane solution (Figs. 3d and 3e).

Supplementary Note 14. Energy evolution during the Au–S bond cleavage in the absence of halogen catalysis

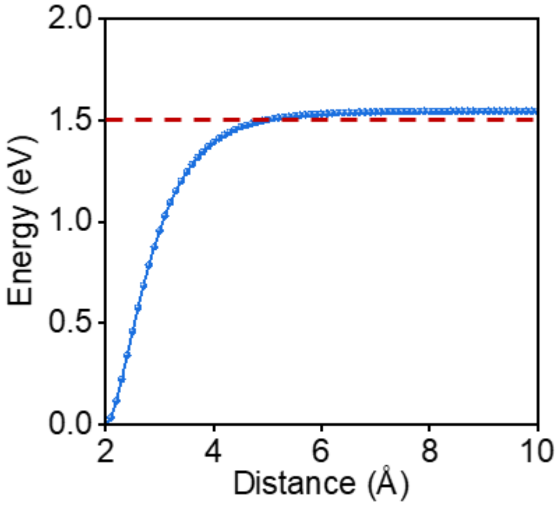

Supplementary Fig. 14 | Energy evolution during the Au–S bond cleavage in the absence of halogen catalysis. The energy needed to break the Au–S bond is guided by the red dash line.

Supplementary Note 15. Evolution of energy and Voronoi charge as the distance between gold tip atoms and chlorine atoms

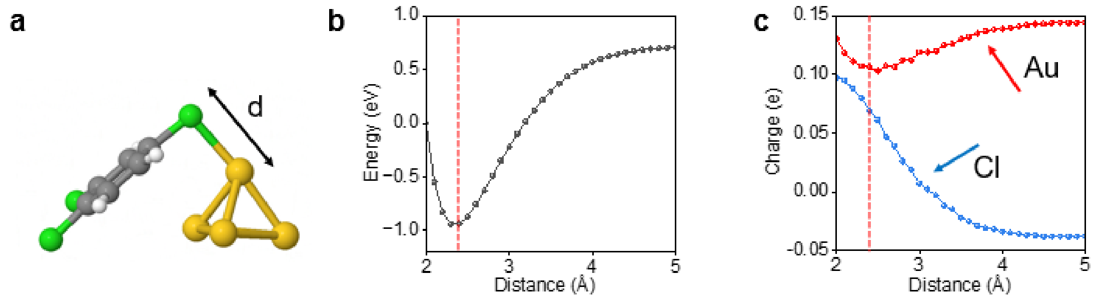

Supplementary Fig. 15 | Evolution of energy and Voronoi charge as the distance between gold tip atoms and chlorine atoms. The optimal distance between gold and halogen atoms is indicated by the red dash line. The positive charge means a loss of electrons.

Supplementary Note 16. Experimental results with the AC–SAc molecule in TBB/TCB at 0.1 V

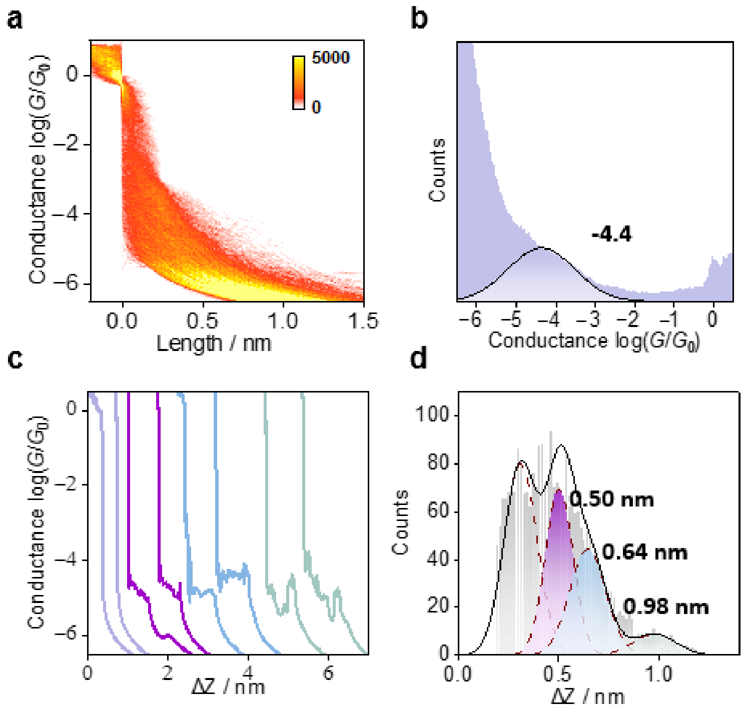

Supplementary Fig. 16 | Experimental results with the AC–SAc molecule in TBB/TCB at 0.1 V. a, 2D conductance-displacement histograms of the AC–SAc molecule. The X axes is divided into 500 parts, that is, bin size of X is 500. While the Y axes is divided into 1000 parts, that is, bin size of X is 1000. Therefore, there are 1000*500 grids. All the single traces are superimposed in this 2D diagram. Whenever a point falls on any grid, the number in that grid will increase by one. The color bar means the number of points in corresponding grid. b, 1D conductance histograms of the AC–SAc molecule. c, Typical single conductance-displacement traces. d, Statistics of conductance plateau lengths for AC–SAc single-molecule junctions in TIB/TCB.

Supplementary Note 17. The discussion of the semiconductive properties of halogen-metal complexes

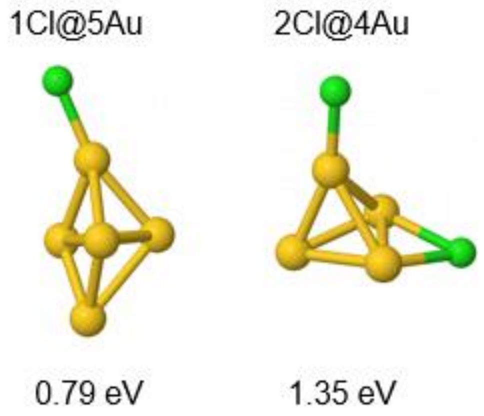

Supplementary Fig. 17 | The HOMO-LUMO gaps of two examples (relaxed halogen-gold complexes). Five gold atoms with one chlorine and four gold atoms with two chlorines are analyzed, respectively. The organo-metallic clusters/complexes generally have relatively large band gaps, which can be proved by optimal measurements[2] and our simulation.

Supplementary Note 18. Theoretical molecular models for energy evolution calculation

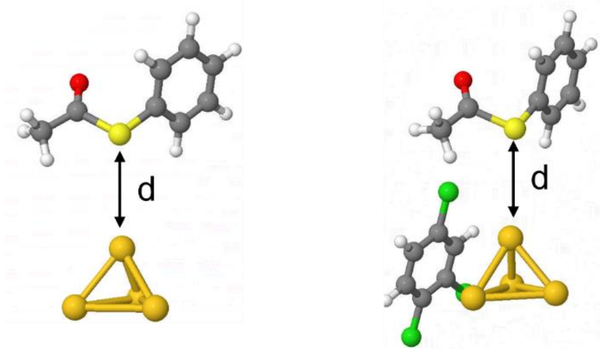

Supplementary Fig. 18 | Molecular models. Molecular models to obtain energy versus distance (d) with and without one solvent molecule as presented in Fig. 4a.

Supplementary Note 19. The final states of halogen atoms (Cl and I) and the topmost gold atom.

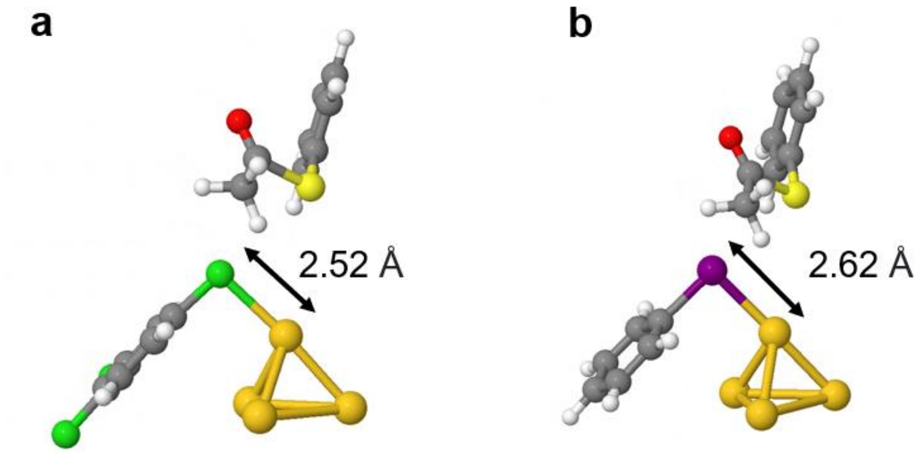

Supplementary Fig. 19 | The final states of halogen atoms (Cl and I) and the topmost gold atom.

Supplementary references

1. Xu, B. Q. *et al.*, Measurements of single-molecule electromechanical properties. *J. Am. Chem. Soc.* **125**, 16164–16165 (2003)

2. Abbas, M. A. *et al.*, Exploring interfacial events in gold-nanocluster-sensitized solar cells: Insights into the effects of the cluster size and electrolyte on solar cell performance. *J. Am. Chem. Soc.* **138**, 390–401 (2015).
