# Supplementary material for: The role of halogens in Au–S bond cleavage for energy-differentiated catalysis at the single-bond limit
Source: Nat Commun. 2023 Nov 24;14:7695. doi: 10.1038/s41467-023-43639-8 (PMC10673828; doi:10.1038/s41467-023-43639-8)
Supplement: Supplementary file 3 — Reporting Summary [file 41467_2023_43639_MOESM3_ESM.pdf]

nature portfolio

Corresponding author(s): Xuefeng Guo Chuancheng Jia Colin J. Lambert Zhongbo Yu

Last updated by author(s): Nov 2, 2023

Reporting Summary

Nature Portfolio wishes to improve the reproducibility of the work that we publish. This form provides structure for consistency and transparency in reporting. For further information on Nature Portfolio policies, see our Editorial Policies and the Editorial Policy Checklist.

Statistics

For all statistical analyses, confirm that the following items are present in the figure legend, table legend, main text, or Methods section.

n/a	Confirmed	
☐	☒	The exact sample size (*n*) for each experimental group/condition, given as a discrete number and unit of measurement
☐	☒	A statement on whether measurements were taken from distinct samples or whether the same sample was measured repeatedly
☒	☐	The statistical test(s) used AND whether they are one- or two-sided<br>*Only common tests should be described solely by name; describe more complex techniques in the Methods section.*
☐	☒	A description of all covariates tested
☐	☒	A description of any assumptions or corrections, such as tests of normality and adjustment for multiple comparisons
☐	☒	A full description of the statistical parameters including central tendency (e.g. means) or other basic estimates (e.g. regression coefficient) AND variation (e.g. standard deviation) or associated estimates of uncertainty (e.g. confidence intervals)
☒	☐	For null hypothesis testing, the test statistic (e.g. *F*, *t*, *r*) with confidence intervals, effect sizes, degrees of freedom and *P* value noted<br>*Give P values as exact values whenever suitable.*
☒	☐	For Bayesian analysis, information on the choice of priors and Markov chain Monte Carlo settings
☐	☒	For hierarchical and complex designs, identification of the appropriate level for tests and full reporting of outcomes
☒	☐	Estimates of effect sizes (e.g. Cohen's *d*, Pearson's *r*), indicating how they were calculated

Our web collection on statistics for biologists contains articles on many of the points above.

Software and code

Policy information about availability of computer code

Data collection	Xtech STMBJ
Data analysis	XMe open-source code (https://github.com/Pilab-XMU/XMe_DataAnalysis)

For manuscripts utilizing custom algorithms or software that are central to the research but not yet described in published literature, software must be made available to editors and reviewers. We strongly encourage code deposition in a community repository (e.g. GitHub). See the Nature Portfolio guidelines for submitting code & software for further information.

Data

Policy information about availability of data

All manuscripts must include a data availability statement. This statement should provide the following information, where applicable:

- Accession codes, unique identifiers, or web links for publicly available datasets
- A description of any restrictions on data availability
- For clinical datasets or third party data, please ensure that the statement adheres to our policy

All data supporting the findings of this study are available within the main manuscript and the supplementary files. Raw data are provided with this paper in the Source Data file. Source data is available at https://zenodo.org/records/10056659.

Research involving human participants, their data, or biological material

Policy information about studies with human participants or human data. See also policy information about sex, gender (identity/presentation), and sexual orientation and race, ethnicity and racism.

Reporting on sex and gender	This study does not involve human research participants.
Reporting on race, ethnicity, or other socially relevant groupings	None.
Population characteristics	None.
Recruitment	None.
Ethics oversight	None.

Note that full information on the approval of the study protocol must also be provided in the manuscript.

Field-specific reporting

Please select the one below that is the best fit for your research. If you are not sure, read the appropriate sections before making your selection.

☐ Life sciences ☐ Behavioural & social sciences ☒ Ecological, evolutionary & environmental sciences

For a reference copy of the document with all sections, see nature.com/documents/nr-reporting-summary-flat.pdf

Ecological, evolutionary & environmental sciences study design

All studies must disclose on these points even when the disclosure is negative.

Study description	We used single-molecule break junction measurements to study the role of halogens in metal catalysis and the effect of catalysis on single bond breaking by monitoring the stretch-induced breaking process, especially the corresponding plateau length in relation to the breaking energy.
Research sample	π-conjugated molecule with acetylthiol terminal groups
Sampling strategy	Over 4000 pieces of STMBJ repeated stretching data were collected
Data collection	In the STMBJ measurement, the distance between the gold tip and the substrate was controlled by a stepper motor and a piezo stack. The bias voltage was applied between the tip and substrate, and the current was used as the feedback to control the movement of the gold tip. During the repeating opening (tip retracting) and closing (tip approaching) cycles, the conductance versus displacement traces was collected, and the traces of the opening cycles were used for further analysis. All measurements were carried out at room temperature.
Timing and spatial scale	In Tianjin, Nankai University, Center of Single-Molecule Sciences Lab, the data have been collected from 2021.2 to 2023.8. Data from the same experiment were collected continuously over a single day. The intervals between different experiments can range from a few days to several months,mainly due to the appointment of experimental instruments and the re-addition of experiments when responding to reviewer comments.
Data exclusions	No data are excluded from the analyses.
Reproducibility	The length splitting phenomenon was repeated in different experimental conditions, and the same phenomenon also appeared in similar systems.
Randomization	The molecule is resolved in the solvent, and the solution was added to the Au substrate electrode randomly.
Blinding	The data was collected totally, without selected.

Did the study involve field work? ☐ Yes ☒ No

Reporting for specific materials, systems and methods

We require information from authors about some types of materials, experimental systems and methods used in many studies. Here, indicate whether each material, system or method listed is relevant to your study. If you are not sure if a list item applies to your research, read the appropriate section before selecting a response.

Materials & experimental systems

n/a	Involved in the study
☒	☐ Antibodies
☒	☐ Eukaryotic cell lines
☒	☐ Palaeontology and archaeology
☒	☐ Animals and other organisms
☒	☐ Clinical data
☒	☐ Dual use research of concern
☒	☐ Plants

Methods

n/a	Involved in the study
☒	☐ ChIP-seq
☒	☐ Flow cytometry
☒	☐ MRI-based neuroimaging

Plants

Seed stocks	None.
Novel plant genotypes	None.
Authentication	None.
